# (How) did attack advertisements increase Affordable Care Act enrollments?

**DOI:** 10.1371/journal.pone.0228185

**Published:** 2020-02-19

**Authors:** Niam Yaraghi, Darrell M. West, Ram D. Gopal, Ram Ramesh

**Affiliations:** 1 Department of Operations and Information Management, University of Connecticut, Stamford, CT, United States of America; 2 Governance Studies, The Brookings Institution, Stamford, CT, United States of America; 3 Governance Studies, The Brookings Institution, Washington, DC, United States of America; 4 University of Warwick, Coventry, England, United Kingdom; 5 SUNY at Buffalo, Buffalo, NY, United States of America; Georgia State University, UNITED STATES

## Abstract

We examine the effects of exposure to negative information in attack advertisements in the context of Affordable Care Act (ACA) and Common Core (CC) education standards and show that they lead to an increase in the ACA enrollments and support of the CC standards. To explain this effect, we rely on the knowledge-gap theory and show that individuals who were exposed to more attack advertisements were also more likely to independently seek information, become more knowledgeable, and consequently support these subjects. In addition to an observational study, to test our hypotheses on the link between exposure to negative information, curiosity, and shifts in knowledge and support levels, we design and conduct a randomized experiment using a sample of 300 unique individuals. Our multi-methods research contributes to marketing literature by documenting a rare occasion in which exposure to attack advertisements leads to increased demand and unveiling the mechanisms through which this effect takes place.

## 1. Introduction

Despite the popular belief, empirical studies on attack advertisements almost unanimously conclude that it hurts everything form box office receipts [1] to brand evaluation [2] and firm value [3]. However, there are some exceptions; dissemination of negative information increases the readership of blogs [4] and sale of bad wines [5]. “Low product awareness” is a major condition identified in the literature under which exposure to attack advertisements leads to increased sales [6]. By analyzing book reviews published in *The New York Times*, Berger et al. [6] show that negative reviews help unpopular authors by increasing their visibility and making more readers aware of their books. This paper contributes to the literature by documenting another example in which dissemination of negative information via attack advertisements by competitors increases demand and uncovering other mechanisms through which this intriguing effect takes place.

This research shows rather than having an immediate effect on consumers’ knowledge, the relationship between exposure to negative information and consumers’ knowledge is mediated by consumers’ curiosity. By analyzing data collected from a series of randomized experiments, we show that exposure to negative information in attack advertisements triggers an individual’s sense of curiosity which in turn will lead to higher knowledge and subsequent adoption of services and products. While we investigate the effects of attack advertisements on enrollment in health insurances provided through Affordable Care Act (ACA), later in the paper we show that our conclusions are not limited to health insurance context and are rather relevant to the domain of negative information dissemination and attack advertisements. To examine if the observed effects have broader implications outside of the case of ACA, we also conduct our experiment using the subject of Common Core (CC) education standards and show that similar mechanisms persist there as well. The consistent results that we observe in both ACA and CC cases corroborate the causal mechanisms that we hypothesize and investigate in this research.

The focus of this paper distinguishes it from prior research by Berger et al. [6] and enables us to examine unique hypotheses on the positive effects of negative information. We discuss these features below.

The difference between anti-ACA advertisements and book reviews is their level of information richness. As we present in appendix, the contents of the anti-ACA advertisements are not informative. They are more rhetorical than substantive. While negative book reviews discuss the reasons for which a book is deemed to be of low quality, anti-ACA advertisements neither mention the details of the ACA nor provide any individually relatable reasons for their attack on ACA. Rather it seems that their producers assumed that public already agrees that ACA is inherently a bad phenomenon and thus there is no need to present them with more arguments against it.

Given these differences, we examine the following mechanism through which attack advertisements about ACA by its opponents, inadvertently, increased ACA enrollment. Building on information-gap theory [7], we hypothesize that exposure to negative information in anti-ACA advertisements triggers the curiosity of individuals and leads them to seek further information through other source to become more knowledgeable about ACA insurances. Although its critics argue that ACA increases the national deficit and imposes an economic burden at social level in the long run, from an individual’s perspective, like other government subsidies, ACA benefits those who take advantage of it in the short run. Consequently, those who know more about subsidized health insurances will be more likely to enroll in one.

Berger et al. [6] uncovered the *moderating* effect of awareness on the relationship between exposure to negative information and purchase decisions. Our work theoretically contributes to the literature by uncovering a *mediated* process through which the relationship between exposure to negative information and purchase decisions is sequentially mediated by curiosity and increased knowledge. Moreover, we show that the effect is not necessarily limited to products with low awareness and can be observed for popular services and products such as ACA and CC.

Another closely related work is the study of Phillips et al. [8] on the voters’ responses to advertisements in 2004 presidential elections. They uncovered four different effects of exposure to negative information disseminated through attack advertisements: reinforcement, backlash, defensive reactance, and position change. Our study not only uncovers information seeking, triggered through curiosity, as an additional effect of exposure to negative information, but also shows that the effects of political advertisements can go beyond their targeted candidate and encompass the phenomena that were not the focus of the advertisement. For example, in the case of ACA we show that the political attack advertisements which were intended to shift voters’ preferences away from the unfavorable candidate, lead to the unintended consequence of increased ACA enrollment, a phenomenon that was not the focus of these attack advertisements.

In this research we undertake two different methods and present two studies. First, in an observational study, we use publicly available datasets on various state level characteristics, ACA enrollment statistics and reports of political advertisements during the 2014 midterm elections to quantify the effect of negative advertisements on states’ ACA enrollment ratios. Second, we test our hypothesis on the mediated effect of exposure to attack advertisements on consumers’ adoption decisions by conducting a randomized experiment on 300 individuals. In this experiment, we compare the effects of exposure to positive and negative advertisements on individuals’ curiosity, their knowledge and subsequent support for two subjects of ACA and Common Core education standards.

The results of our first study indicate that spending on negative advertisements increases the ACA enrollment ratio at the state level. Our second study explains why this effect takes place; our results show that exposure to negative information via attack advertisements triggers individuals to seek further information about the subject of the advertisement, which in turn, increases their knowledge about the subject which subsequently increases their support level.

## 2. Theoretical framework

Ansolabehere et al. [9,10] analyzed the effects of negative campaign advertisements on electorate mobility and showed that such advertisements reduce voters’ turnout and increase their cynicism about public officials. Although their findings corroborate those of Basil et al. [11] who also show that negative advertisements reduce positive attitudes towards candidates in a political race, recent literature in political science challenges their hypothesis and instead predicts a positive or at least natural effect on voters’ turnout. This stream of literature points to the importance of information provided through attack advertisements and argues that this additional information will enable voters to make better decisions and motivates them to participate in elections [12,13]. Krupnikov [14] consolidates these conflicting empirical results by arguing that exposure to negative information demobilize only if it happens after an individual has selected a preferred candidate and the negativity is about this selected candidate.

Soroka (2016) provides a comprehensive discussion of negativity bias and documents various examples in which humans tend to prioritize negative information over positive information [15]. Recent experiments on negativity bias show that there is on average higher activation in response to negative stimuli than to positive ones [16] and negative news elicits stronger and more sustained reactions than positive news [17], other experiments show that women are more attentive than men to negative news content [18]. Interestingly, prior research documents that news consumers have a higher demand for negative news content [19].

The findings of Krupnikov [14] only pertain to subjects that require high cognitive processing effort, such as evaluating political candidates. Prior research documents the *primacy* effect and shows that to form an opinion, individuals more heavily rely on information they receive early on [20], unless the subject requires the individuals to devote high processing efforts, in which they would hold judgment until they have received and processed all the information [21,22]. Ein-Gar et al. [23] uncover the *blemishing* effect which bolsters and intensifies the primacy effect. They show that when a subject requires low processing effort and individuals are first presented with positive information based on which they have already created a positive opinion about the subject, presenting them with minor negative information would accentuate rather than attenuate that initial positive impression. In this research we examine the effects of exposure to positive and negative information on individuals’ opinions about subjects that require high cognitive processing. One would expect that in these situations, since individuals hold judgment until all information are processed, the sequence in which they receive the information should not affect their judgment and more importantly, receiving negative information should negatively affect their overall impression of the subject. However, we uncover that when individuals are first provided with negative and rhetorical messages about a potentially beneficial subject, they are more likely to independently seek further information about it, and therefore are more likely to form a positive impression of the subject.

Political scientists and marketing researchers have examined cases in which the advertisements have produced uptake beyond the intended product or audience. For example, Urban and Niebler [24] examine the effects of political advertisements on campaign donations in bordering areas of noncompetitive states that receive spillover advertisements from competitive states. They show that the aggregate campaign donations from zip codes in non-contested states which were exposed to political ads was substantially more than in similar zip codes without advertisements. Other researchers have shown that unintended exposure of individuals to advertisements has a strong impact on their level of persuasion [25]. Using the television advertisements for antidepressants, Shapiro [26] shows that advertisements by one company leads to increased sales of similar products by rival companies. Interestingly, Sinkinson and Starc [27] observe a similar phenomenon in direct to consumer advertisements for anti-cholesterol drugs. They show that such advertisements by one company increases the sale of drugs of the non-advertised competitors in the same class.

The current study contributes to the literature by investigating the unintended side-effects of exposure to attack advertisements on voters’ decision to independently seek further information about the subjects mentioned in the advertisements. Our study shows that the impact of advertisements is not limited to voting decisions and may further extend to individuals’ curiosities and perceptions about other contents of such advertisements.

Information-gap theory [7] posits that curiosity—manifested as the desire to seek knowledge—is triggered when an individual is presented with a manageable knowledge gap. As we present in the appendix, political attack advertisements which were aired during the Congress’s midterm elections heavily criticized ACA without providing substantial information to support their arguments against it. Repeatedly attacking a concept and portraying it as a negative phenomenon without providing supporting information triggers curiosity by making individuals to feel a knowledge-gap between what they know and what they want to know. These arguments are further supported by research that shows exposure to curiosity-evoking advertisements turns an individual from a passive information processor to an active information seeker [28] and results in greater elaboration and information search as well as better learning of information [29]. The knowledge gap that is created by anti-ACA advertisements is fairly manageable thanks to the wealth of easy to understand information resources about ACA. The education level of uninsured -whom ACA is specifically designed for-is very low. According to the statistics of Economic Research Initiative on the Uninsured (ERIU), 56.3 percent of uninsured Americans have a high school diploma as their highest level of education and among uninsured adults born outside the US, 73.7 percent have at most a high school diploma.

Given this fact, ACA advocates designed information resources and marketing materials to specifically inform an audience with little or no formal schooling and thus we argue that the knowledge gap that results from exposure to anti-ACA advertisements will be fairly easy to fill. Therefore, we hypothesize that anti-ACA advertisements create a manageable knowledge-gap and lead their audience to fill it by independently seeking further information. Those who seek more information will consequently know more about the benefits of ACA’s subsidized health insurance plans and are thus more likely to enroll in one.

Our hypothesis is consistent with the models proposed by Mayzlin et al. [30]. In their theoretical work, they show that “there exists an equilibrium where the high-quality firm chooses to produce “empty messages devoid of any attribute information in order to invite the consumer to engage in search, which is likely to uncover positive information about the product.” Our research not only tests the theory in an empirical setting with real-world data, but also by shows that the effect is not limited to “empty” positive advertisements that are run by the firms themselves to increase adoption, but rather extents to the empty negative advertisements that run by opponents to decrease adoption or support of an unfavorable subject or candidate, respectively.

We can formally present our hypotheses as follows:

- *H1*: *Exposure to negative information is positively associated with curiosity*.
- *H2*: *Curiosity is positively associated with increased knowledge about the subject*.
- *H3*: *Knowledge about the desirable features of the product is positively associated with adoption*.

## 3. Study one: Effects of anti-ACA advertisements on states’ enrollment ratios

Lerman et al. [31] examine how individuals’ decision to enroll in ACA insurances are affected by their political beliefs. In a field experiment, they assigned uninsured individuals to two groups. The first group was asked to sign up for ACA insurances through a private website while the other was tasked with signing up for the insurances through ACA’s governmental website. While the rate of enrollment was equal in the two groups, Republicans were significantly more likely to signup if they were assigned to the private website. Such partisan differences in perceptions about ACA are also observed by Fowler et al. [32]. They analyze the relationship between exposure to local news and advertisements about ACA and the individuals’ perception on how informed they were about and how favorable they were toward ACA. Although they did not find any differences in the relationships between exposure to information and the perception of being informed about ACA by political party, they report that exposure to news media and advertisements lead to higher favorability toward ACA among Democrats. Our second study consolidates these findings by showing how curiosity mediates the relationship between exposure to advertisements, information about, and favorability towards ACA.

In another closely related research, Gollust et al. [33] show that positive advertisements about ACA leads to higher rates of enrollment while attack advertisements lead to opposite effects. The difference in their findings and those of ours could possibly be due to the differences in methodological approaches; we extend their work by explicitly dealing with the endogeneity of advertisements. Airing of attack advertisements are informed decisions that are made by political candidates with careful consideration of the audience and their potential impact. Since these advertisements are not randomly distributed among different geographical regions in the sample, they should be examined within a modeling framework that incorporates their endogeneity. Our study sheds light on our understanding of the effects of such advertisements by considering them as endogenous variables and parsing out their actual effects from those that may have been confounded by other unobserved variables.

### 3.1. Data

In this observational study, we use state-level data on anti-ACA advertisements and ACA enrollment ratios as well as multiple state-level characteristics to estimate the effects of the anti-ACA advertisements on enrollment. We also collect data on twelve variables that broadly characterize the states’ political and economic landscapes as well as the features of their health insurance markets before and after ACA implementation. The data is collected from multiple publicly available sources including Kaiser Family Foundation (KFF), Centers for Medicaid and Medicare Services (CMS), Department of Health and Human Services (HHS), US Election Atlas, Yahoo Finance, US Census, Wesleyan Media Project (WMP) [34] and the Association of Religion Data Archives (ARDA). Table 1 presents the list of the variables used in this study along with their definitions and sources. Data on ACA enrollment includes those who signed up between November 15, 2014 and February 15, 2015 (the open enrollment period). We use the WMP codebook to identify the topic of political advertisements. The anti-ACA ads include the total number of advertisements run by or for Republican candidates for either House or Senate in each state through the primary and general elections in year 2014 which mention either “Prescription Drugs”, “Health Care”, “Affordable Care Act”, “Obamacare” or “Health Care Law”. Similarly, the pro-ACA ads include the total number of ads that mention similar issues but are run by Democrat candidates. Although some media markets span between multiple states, we use the state in which the election took place to aggregate the observations in our dataset. For example, if an ad for a candidate for a Senate seat in state A was aired in a media market that covers two states of A and B, we count that ad only for state A which was the state in which the race took place, as coded in the Wesleyan Media Project dataset. It is important to note that while ACA continued over the following years, the anti-ACA advertisements stopped after the elections in 2014. Because the independent variable (Anti-ACA ads) exist only in a single year, it was impossible to undertake a panel data analysis. For all other variables, we have collected the most recent available data in February 2015. Note that since these variables are at state levels, their values would not change within a span of one year. In the appendix, we provide a detailed explanation for including each of these variables in our analysis. Descriptive statistics of these variables are presented in Table 2.

**Table 1:** Descriptions and data sources of state-level variables.

Variable	Description	Source	URL
Enrollment count	Enrollment numbers at the end of the second open enrollment period. This includes number of individuals who have selected a marketplace plan through both state- and federal-based exchange systems and excludes those who were enrolled through Medicaid and CHIP programs	HHS	https://aspe.hhs.gov/system/files/pdf/83656/ib_2015mar_enrollment.pdf
ACA market size	Total number people between ages 0 and 64 who were either uninsured or had private insurance	KFF	http://kff.org/other/state-indicator/total-population/
Enrollment ratio	The ratio of enrollees to ACA market size	Authors’ calculation	N/A
Young Invincibles	Number uninsured people between ages 18 to 34 divided by the total number of uninsured people	CMS	https://data.cms.gov/dataset/The-Number-of-Estimated-Eligible-Uninsured-People-/pc88-ec56
Low Income	Number of uninsured people with income below 134% of federal poverty level divided by the total number of uninsured people	CMS	https://data.cms.gov/dataset/The-Number-of-Estimated-Eligible-Uninsured-People-/pc88-ec56
ACA premium	Average premium for lowest cost silver, second lowest cost silver and lowest cost bronze plans (log transformed)	HHS	http://aspe.hhs.gov/health/reports/2013/MarketplacePremiums/ib_premiumslandscape.pdf
Private Insurance Premium	Average per person monthly premiums in the individual market (log transformed)	KFF	http://kff.org/other/state-indicator/individual-premiums/
Uninsured Females	Number of uninsured females divided by the total number of uninsured people	CMS	https://data.cms.gov/dataset/The-Number-of-Estimated-Eligible-Uninsured-People-/pc88-ec56
Uninsured Latinos	Number of uninsured Latinos divided by the total number of uninsured people	CMS	https://data.cms.gov/dataset/The-Number-of-Estimated-Eligible-Uninsured-People-/pc88-ec56
Liberal voters	Percentage of votes for Barak Obama in 2012 presidential elections	US Election Atlas	http://uselectionatlas.org/RESULTS/data.php?year=2012&datatype=national&def=1&f=0&off=0&elect=0
Insurance cancelations	Number of cancelled insurances divided by the total number people with private insurance	Yahoo! Finance	http://finance.yahoo.com/news/policy-notifications-current-status-state-204701399.html just in case: washington had 92% of cancelations!
Catholic church members	Number of adherents in 2010 divided by the non-elderly population	ARDA	http://www.thearda.com/Archive/Files/Downloads/RCMSST10_DL2.asp
Education	Percent of population with a Bachelor's degree or higher in 2009	US Census	http://www.census.gov/compendia/statab/cats/education/educational_attainment.html
Anti ACA ads	Number of political ads in 2014 run by Republican candidates for either House or Senate that mention either “Health Care”, “Affordable Care Act”, “Obamacare” or “Health Care Law”.	WMP	http://mediaproject.wesleyan.edu/ (Available through WMP for a fee)
Pro ACA ads	Number of political ads in 2014 run by Democrat candidates for either House or Senate that mention either “Health Care”, “Affordable Care Act”, “Obamacare” or “Health Care Law”.		
State-run exchange	Equals to one for states that created their own Health insurance marketplaces	KFF	http://kff.org/health-reform/state-indicator/health-insurance-exchanges/#
Medicaid expansion	Equals to one for states that expanded their Medicaid program	KFF	http://kff.org/health-reform/state-indicator/state-activity-around-expanding-medicaid-under-the-affordable-care-act/
Congress’s midterm competitiveness index (*CI*)	Competitive index for Congress midterm elections	Cook Political Report	http://cookpolitical.com/house/charts/race-ratings and http://cookpolitical.com/senate/charts/race-ratings
Population (**Logpop**)	State population (log transformed)	US Census Bureau	http://factfinder.census.gov/faces/nav/jsf/pages/index.xhtml

**Table 2:** Mean, standard deviation and Pearson inter-correlations of state level variable.

Variable	Mean	Std. Dev.	1	2	3	4	5	6	7	8	9	10	11	12	13	14	15	16	17	18
1.Enrollment count	229178	328676																		
2.ACA market size	1006447	1336935	0.95758 (< .0001)																	
3.Enrollment ratio	0.22319	0.07726	0.18462 (0.1947)	0.04497 (0.7540)																
4.Young Invincibles	0.39875	0.02795	-0.16522 (0.2466)	-0.11399 (0.4258)	-0.25549 (0.0704)															
5.Low Income	0.49426	0.05846	0.15841 (0.2669)	0.17658 (0.2152)	-0.37113 (0.0073)	0.26328 (0.0619)														
6.ACA premium (log)	5.69575	0.17724	-0.03485 (0.8082)	-0.07937 (0.5798)	0.29579 (0.0351)	-0.11786 (0.4101)	-0.20888 (0.1413)													
7.Private Insurance Premium (log)	5.57839	0.52676	0.26080 (0.0645)	0.36836 (0.0078)	-0.02679 (0.8519)	0.05072 (0.7237)	-0.16961 (0.2341)	0.16676 (0.2422)												
8.Uninsured Females	0.44318	0.02719	0.19084 (0.1798)	0.20340 (0.1523)	-0.33388 (0.0166)	-0.02926 (0.8385)	0.29578 (0.0351)	-0.08869 (0.5360)	-0.20700 (0.1450)											
9.Uninsured Latinos	0.14634	0.12739	0.52338 (< .0001)	0.60910 (< .0001)	-0.18628 (0.1906)	-0.05300 (0.7119)	0.12358 (0.3876)	-0.19271 (0.1755)	0.41990 (0.0022)	-0.03938 (0.7838)										
10.Liberal voters	0.48977	0.11801	0.03759 (0.7934)	0.03233 (0.8218)	0.22262 (0.1164)	0.11613 (0.4171)	-0.09541 (0.5054)	0.04404 (0.7589)	0.27162 (0.0538)	-0.55477 (< .0001)	0.15921 (0.2644)									
11.Insurance cancelations	0.25934	0.28759	0.09850 (0.4917)	0.07955 (0.5790)	-0.05354 (0.7091)	0.08621 (0.5475)	0.08530 (0.5518)	-0.03003 (0.8343)	-0.15173 (0.2879)	0.15821 (0.2675)	0.04843 (0.7357)	0.07650 (0.5937)								
12.Catholic church members	0.27710	0.17068	0.03472 (0.8089)	0.06545 (0.6482)	0.08648 (0.5462)	0.28059 (0.0461)	-0.21576 (0.1284)	0.10148 (0.4786)	0.42770 (0.0017)	-0.49368 (0.0002)	0.38362 (0.0055)	0.46819 (0.0005)	-0.02667 (0.8526)							
13.Education	0.27590	0.05555	-0.03004 (0.8342)	-0.01946 (0.8922)	0.19852 (0.1626)	0.07600 (0.5961)	-0.37290 (0.0070)	-0.07457 (0.6030)	0.22212 (0.1172)	-0.49567 (0.0002)	0.21687 (0.1264)	0.73279 (< .0001)	0.14654 (0.3048)	0.42074 (0.0021)						
14.State-run Exchange	0.33333	0.47610	-0.10331 (0.4707)	-0.05148 (0.7198)	-0.05944 (0.6786)	0.19300 (0.1748)	0.06108 (0.6702)	-0.15105 (0.2900)	0.34303 (0.0137)	-0.39565 (0.0041)	0.31171 (0.0260)	0.54015 (< .0001)	0.31589 (0.0239)	0.33249 (0.0171)	0.48446 (0.0003)					
15.Medicaid Expansion	0.52941	0.50410	-0.18971 (0.1824)	-0.11956 (0.4034)	-0.19325 (0.1742)	0.29056 (0.0386)	-0.03913 (0.7852)	-0.10036 (0.4835)	0.28231 (0.0447)	-0.40436 (0.0033)	0.23296 (0.0999)	0.56307 (< .0001)	0.18173 (0.2018)	0.43905 (0.0013)	0.36754 (0.0080)	0.58333 (< .0001)				
16. Competitiveness index	6.31373	5.89403	0.37943 (0.0060)	0.36768 (0.0079)	0.03577 (0.8032)	0.06519 (0.6495)	0.04973 (0.7289)	0.04204 (0.7696)	0.17569 (0.2175)	0.01075 (0.9403)	0.22243 (0.1167)	-0.02253 (0.8753)	0.37359 (0.0069)	0.15573 (0.2752)	-0.09556 (0.5048)	0.04752 (0.7406)	0.13146 (0.3578)			
17.Population (log)	14.61613	1.04016	0.72232 (< .0001)	0.75868 (< .0001)	-0.05239 (0.7150)	0.00119 (0.9934)	0.27302 (0.0526)	-0.27729 (0.0488)	0.26274 (0.0625)	0.18083 (0.2041)	0.43063 (0.0016)	0.05042 (0.7253)	0.14112 (0.3233)	0.09607 (0.5025)	-0.02395 (0.8675)	-0.03326 (0.8168)	-0.01334 (0.9260)	0.23431 (0.0979)		
18. Anti-ACA ads	5877	6508	0.22681 (0.1095)	0.16591 (0.2446)	-0.08374 (0.5591)	0.06538 (0.6485)	0.25737 (0.0683)	-0.06217 (0.6647)	-0.14627 (0.3057)	0.42313 (0.0020)	-0.12296 (0.3900)	-0.26107 (0.0643)	0.15132 (0.2891)	-0.31259 (0.0255)	-0.33505 (0.0162)	-0.28430 (0.0432)	-0.09678 (0.4993)	0.07617 (0.5952)	0.22812 (0.1074)	
19. Pro-ACA ads	1192	1527	0.34197 (0.0140)	0.28906 (0.0397)	-0.05496 (0.7017)	-0.14348 (0.3152)	0.13568 (0.3425)	0.10534 (0.4619)	0.06141 (0.6686)	0.17392 (0.2222)	0.04668 (0.7450)	-0.08403 (0.5577)	0.11133 (0.4367)	-0.08962 (0.5317)	-0.20038 (0.1586)	-0.23463 (0.0975)	-0.00990 (0.9450)	0.17949 (0.2076)	0.23323 (0.0995)	0.72047 (< .0001)

The p-value of correlations are reported in parentheses

To calculate the Congress’s midterm competitiveness index (CI), we used Cook Partisan Voting Index (Cook PVI). PVI groups the political race in a congressional district into the following four categories based on how strongly it leans toward the Democratic or Republican Party, compared to the nation as a whole.

- Solid: These are the districts that strongly lean towards Democratic or Republican Party and thus candidates in these districts do not face tangible competition from those of the other party.
- Likely: These districts are not considered competitive but have the potential to become engaged.
- Lean: These are considered competitive races, but one party has an advantage.
- Toss-Up: These are the most competitive; either party has a good chance of winning.

We assign a score of 1 to solid districts (which have the least competitive races), 2 to likely districts, 3 to lean districts, and 4 to toss-up districts (which have the most competitive races). Multiple districts within a state may be running midterm elections for House of Representatives. In these cases, we add the scores of all the districts within a state. We use the same scoring system for the Senate midterm elections as well (the only difference is that Senate races are be at state level). We calculate our final Competitiveness Index (CI) as the sum of the scores for the elections of both House and Senate at each state.

### 3.2. Method

In order to correctly estimate the effects of anti-ACA advertisements on states’ enrollment ratios, three empirical issues need to be addressed. First, while the number of observations is relatively small, the number of state-level control variables is very large; many of these variables may be driven by the same unobserved factors and thus could be highly correlated with each other. Consequently, keeping all of the control variables in the model will significantly reduce the model’s degrees of freedom. To overcome this issue, we conducted a Principal Component Analysis (PCA) and reduced the number of control variables into a set of components which best explain the variations in our dataset. The details about the PCA are available in the appendix. Second, the dependent variable is bounded between the interval of zero and one. To overcome this issue, we provide an econometric specification in which the dependent variable follows a *beta* distribution and use maximum likelihood method to estimate our model. The details of the model specification are available in the appendix. Third, the anti-ACA advertisements are potentially endogenous and rather than being randomly distributed, are driven by factors outside of our model. We use the competitiveness of Congress’s midterm elections as an instrumental variable to adjust for the endogeneity of the anti-ACA advertisements.

To instrument for the anti-ACA advertisements, we propose to exploit the heterogeneity among the competitiveness of the midterm elections for the US Senate and the House of Representatives. An appropriate instrumental variable should satisfy two conditions. First, it should have no partial effect on the dependent variable (exogeneity). Second, it should be related with the endogenous explanatory variable (relevance). In the following, we first provide a rationale for the choice of competitiveness of the midterm elections as an instrument and then empirically examine our arguments in Section 3.3.

As we show in the appendix, anti-ACA advertisements were a part of larger political campaigns during the Congress’s midterm elections. In the swing states such as Kentucky, Arkansas, Louisiana, and North Carolina where the midterm elections were more competitive, spending on anti-ACA advertisements was much higher, and thus we expect the relevance condition to hold. On the other hand, the competitiveness of the midterm elections should not affect the ACA enrollment ratio which means that the exogeneity condition is also expected to hold. This makes the competitiveness of the midterm elections an attractive choice for an instrumental variable.

### 3.3. Results

The results of the principal component analysis are shown in S3 Tables in the S1 Appendix. We only retain the first four components since their eigenvalues are higher than one (thus *j*∈(1,2,3,4)). Note that the components with an eigenvalue of less than one account for less variance than did the original variable and so are of little use. These four components explain about 67% of the total variance in our dataset. We consider the four component scores (*C*_*ij*_) as our main control variables in the subsequent analyses (as vectors of ***X*** and ***W*** matrices in equation (1) in the appendix). The rotated factor matrix along with the scree and variance plots is presented in S2 Table and S2 Fig in the S1 Appendix.

To correct for the endogeneity of anti-ACA advertisements, we follow the classic two stage method. In the first stage, we run an Poisson regression in which the dependent variable is the number of anti-ACA advertisements and the right hand side variables include the instrumental variable (competitiveness of midterm elections, *CI*) along with the four principle components (*PC1*,*…*, *PC4*). As shown in the last column of Table 3, the instrument is highly significant (*p-value<0*.*0001*) and positively associated with the spending on the anti-ACA advertisements. This implies that the competitiveness index, *CI*, satisfies the relevance assumption. That is, Cov (*AntiACA Ads*, *CI*) ≠ 0.

**Table 3:** Anti-ACA ads as a function of CI, and other controls.

Variable	Anti-ACA ads	
	(1)	(2)
CI	0.013*** (0.001)	0.016*** (0.003)
PC1		-0.514*** (0.002)
PC2		-0.142*** (0.002)
PC3		0.218*** (0.002)
PC4		0.144*** (0.002)
Constant	8.593*** (0.003)	8.420*** (0.003)

*P<0.1; **p<0.05

***p<0.001

In the second stage, we use the estimates of spending on anti-ACA advertisements from the first stage, as a vector of ***X*** and ***W*** matrices. Further empirical details are presented in the appendix. The estimation results are presented in Table 4. Note that in this table, number of pro and anti- ACA ads are scaled to 1000. The first three columns do not correct for endogeneity of the anti ACA advertisements; this implies that ***X*** and ***W*** matrices include the original values of anti-ACA spending rather than its estimates from the first stage. The last three columns represent the maximum likelihood estimation results after correcting for endogeneity of the anti-ACA advertisements. As shown in column (6) of Table 4, the coefficient of anti-ACA advertisements is estimated as 0.144. This is the unbiased estimate of the coefficient of anti-ACA advertisements and is significant and positive. Note that without correcting for endogeneity of *Anti-ACA Ads*, the model will result in underestimation of the coefficient.

**Table 4:** Beta regression estimation results of equation (1).

Variable	1 Without IV	2 Without IV	3 Without IV	4 IV(2^nd^ stage)	5 IV(2^nd^ stage)	6 IV(2^nd^ stage)
***Location Submodel (estimates of β)***						
Anti ACA ads (×1000)	-0.005 (0.009)	0.005 (0.009)	0.016 (0.012)	-0.022 (0.020)	0.144*** (0.039)	0.144*** (0.039)
Pro-ACA ads (×1000)			-0.058 (0.049)	-0.012 (0.039)		-0.011 (0.030)
PC1		0.101* (0.055)	0.111* (0.055)		0.425*** (0.102)	0.421*** (0.102)
PC2		-0.078 (0.055)	-0.064 (0.056)		0.034 (0.058)	0.035 (0.058)
PC3		-0.126** (0.052)	-0.137** (0.052)		-0.267*** (0.061)	-0.267*** (0.060)
PC4		-0.157** (0.050)	-0.174** (0.051)		-0.298 (0.060)	-0.298*** (0.059)
Constant	-1.214 (0.079)	-1.290 (0.073)	-1.287*** (0.072)	-1.101*** (0.133)	-2.115*** (0.239)	-2.099*** (0.242)
***Fit statistics***						
-2ln L	-123.59	-140.21	-141.59	-124.71	-151.27	-151.40
AIC	-117.59	-126.21	-125.59	-116.71	-137.27	-135.40

*P<0.1

**p<0.05

***p<0.001

To test the robustness of our results, we consider an alternative way of counting the political advertisements in media markets that span multiple states and count the political advertisements in such markets for all of the states that they span. The results remain consistent with those presented in Table 4.

### 3.4. Limitations

In the following, we discuss the limitations of the first study and explain how we overcome them in the next study. The number of observations is restricted to the 50 states and the District of Columbia. Given the lack of granular datasets, we are not able to increase our sample size. This reduces the statistical power in our estimates and therefore, it will be very difficult to draw causal conclusions from its findings. We could not run the analysis at a more granular level such as media market area because enrollment data at more granular levels are only available for 37 states that did not run their own health insurance exchange platform and instead used Healthcare.gov. Additionally, at the media market level analysis we would lose even more data because many of the control variables that are used in the main analysis are only available at state level.

To address this concern, we collected data about as many different state level characteristics as we could in order to control for as much variation as possible. We also used instrumental variables and estimated our model using 2SLS method to adjust for the endogeneity of the anti-ACA advertisements. Despite these remedies, we acknowledge that since our data are at state level, causal inferences cannot be made. We conduct the first study only to observe the effects of negative advertisements on ACA enrollments, and then use these observations to guide us in the second study in which we carefully test our hypotheses using individual level data collected through various experiments. In other words, the first study shows us what the effect of negative advertisements was while the second study helps us explain why such effect took place.

## 4. Study two: Effects of information exposure on individuals’ curiosity, increased knowledge and increased support

As discussed earlier, we hypothesize that exposure to anti-ACA advertisements positively affects the individuals’ decision to adopt the service or product through the following mechanism. Exposure to negative information creates a sense of curiosity which entices individuals to learn more about the service through various resources such as conversing with others, reading articles and listening to others’ discussion about it. This will lead to increased awareness about the existence and the benefits of health insurance for individuals. Ceteris paribus, those who are more aware of ACA health insurances are more likely to purchase them.

### 4.1. Method

In the following, we describe the design of the experiment which consists of six consecutive steps. At the first step, we randomly assign individuals to three groups that are respectively named positive, negative and control.

At the second step, in all of the groups, we measure the baseline knowledge of the individuals about the ACA by asking them to take a short quiz which includes five questions. The level of their knowledge is determined in a 0 to 5 scale based on how they score in the quiz. We also ask them to express how much they support ACA in a 5-point Likert scale.

In the third step, we respectively show individuals in positive and negative groups a 30 second positive or negative advertisement video about ACA. This video is the treatment in our experiment. The individuals in control group do not watch any advertisement video. Both the positive and negative advertisement videos are actual advertisements that were run during the mid-term elections and are retrieved from YouTube.com.

In the fourth step, we present individuals with four different topics and ask them to select the option which they are interested to know more about. ACA was one of the topics, the remaining three topics were not relevant to ACA and focused on other issues such as education policy and lesser known politicians. We measure their curiosity, as a binary variable, based on this selection. If the individual opts to know more about ACA, then we consider her to be curious about the topic. If she chooses one of the other three topics, then we consider her incurious. The reason that we provide more options to users is because we assumed that if users were only given the option to whether or not learn more about ACA, they will be more susceptible to priming effect as compared to a situation where they have more options and can choose from multiple subjects. We remove the potential noise by operationalizing the curiosity as a binary variable which is equal to one only if a user has chosen to learn more about ACA and zero if any of the other nonrelated subjects were selected.

In the fifth step, based on their selection in the previous step, we present individuals a simple paragraph (about 5 sentences) which includes information about the topic of their choice. The individuals could find the answer to all of the quiz questions in the information provided about the ACA, had they chosen to know more about this topic.

Finally, after they have read the information piece, in the sixth step we ask individuals to take the quiz again to measure their knowledge post treatment. The questions in the quiz are identical to the ones that were asked at the beginning of the experiment. We also ask them to express their level of support about ACA again.

To examine the role of prior knowledge and personal opinions of individuals on the effect of the advertisements, we repeat our experiment on Common Core (CC) education standards instead of ACA. Common Core education standards constitute a much less controversial issue as compared with ACA and thus the political opinions and convictions of respondents may play a less salient role on how they respond to the questions in the experiment. Examining CC in addition to ACA, also broadens the focus of this study and adds to the generalizability of our findings.

This design allows us to study how exposure to a certain kind of advertisement triggers an individual’s curiosity and how it could subsequently lead to increased knowledge about and support of the advertisement’s subject. More importantly, the experiment design allows us to estimate the causal effects of multiple mediator variables. Because we have measures of knowledge and support (or enrollment) before and after exposure to different types of advertisements, we can attribute the differences in the curiosity and the subsequent shifts in measures of knowledge and support to differences in advertisement treatment.

For both topics (ACA and CC), we conduct our experiment on samples from populations inside and outside of the US. Because both ACA and CC are topics that are more discussed in national politics, individuals outside of the US may be less educated about these topics as compared with the individuals who live in the US. Therefore the sample of individuals outside of the US will serve as a proxy for low information voters. Also, individuals outside of the US are much less likely to have a viewpoint about these topics based on their political party affiliations. Given the differences in information and political convictions between those who live in the US and their international counterparts, it is interesting to examine how exposure to advertisements affects these two samples differently. We thus have twelve samples: 2 topics (ACA, CC) × 3 treatment levels (positive, negative, no advertisement) × 2 populations (US and international). We used Amazon Mechanical Turk to recruit 300 individuals to participate in our study.

We used the following videos as treatments for positive and negative advertisements about ACA and CC.

- Pro-ACA: https://www.youtube.com/watch?v=8NKVkJE0xRA
- Anti-ACA: https://www.youtube.com/watch?v=0N-GF-XDVIM
- Pro Common Core: https://www.youtube.com/watch?v=moOBWJYSI_U
- Anti-Common Core: https://www.youtube.com/watch?v=j2_NPN2eI6E

### 4.2. Results

Sample characteristics of participants in ACA and CC experiments are presented in S5 and S6 Tables in the S1 Appendix, respectively. In each table, the left panel shows the characteristics of the sample drawn from the population inside the United States and the right panel shows the characteristics of the sample drawn from the population outside of the United States. We can observe that the variables in each of the three arms of the experiment have very similar distributions. This confirms that the subjects are randomly assigned to the experiment groups.

We first present descriptive statistics on the effects of the advertisement type on three outcomes of curiosity, increase in knowledge and increase in support among the individuals sampled from populations inside and outside of the US. While curiosity, increase in knowledge and increase in support are all higher in the group of individuals who were exposed to negative advertisements for both topics of ACA and CC, this difference is only statistically significant in some of the cases as detailed below. Distributions of baseline knowledge and support for ACA and CC among the two samples of individuals inside and outside of the US are presented, respectively, in S3 and S4 Figs at the S1 Appendix.

**Fig 1:**
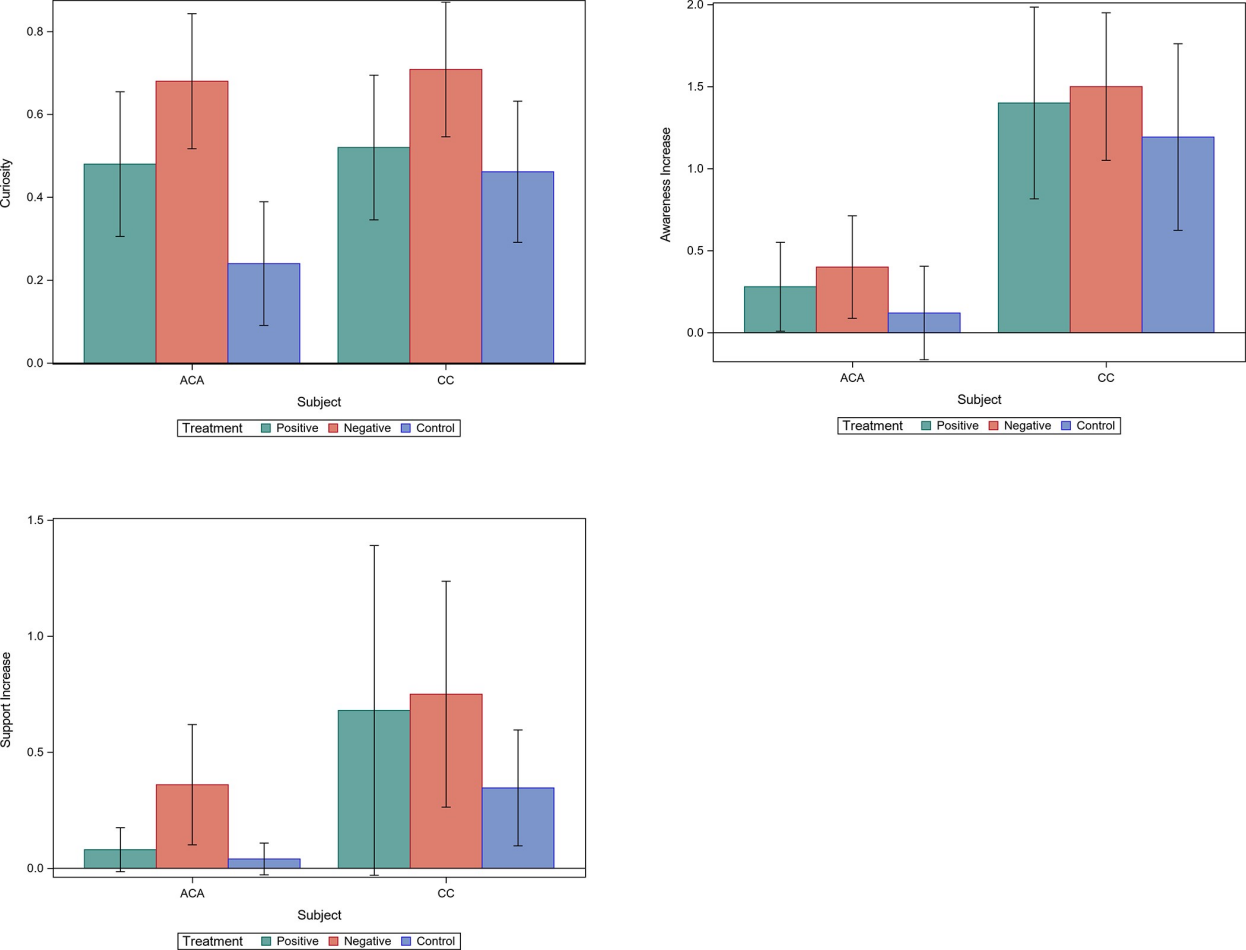


**Fig 2:**
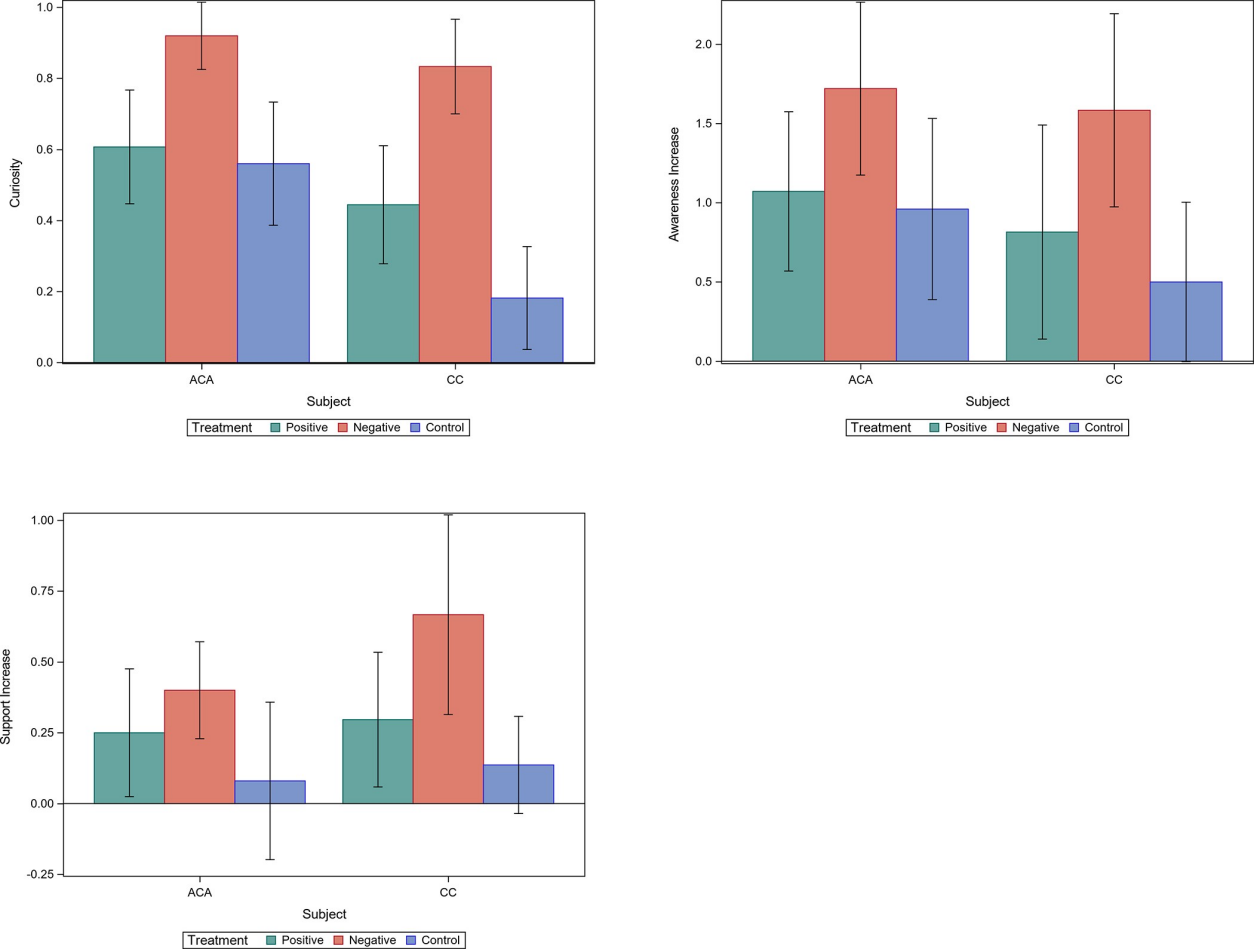


Fig 1A, 1B and 1C 1 shows the effects of advertisements on the sample of individuals in the US on respectively their curiosity, awareness increase and support increase. For the ACA topic, the analysis results confirm the effects of advertisement type on curiosity (*F*_(74,2)_ = 5.38,*p*<0.01) and increase in support (*F*_(74,2)_ = 3.30,*p*<0.05). Fig 2A, 2B and 2C shows the effects of advertisements on the sample of individuals outside of the US on respectively their curiosity, awareness increase and support increase. For the ACA topic, only the mean of curiosity varies significantly among the three groups (*F*_(75,2)_ = 4.95,*p*<0.01). For the common core standards topic, both the curiosity (*F*_(75,2)_ = 13.12,*p*<0.0001) and the increase in support (*F*_(75,2)_ = 2.95,*p*<0.1) are significantly different among the three groups of the advertisements.

While through the descriptive statistics we can observe the differences among treatment groups, we cannot examine the interplay between the concepts of curiosity and shifts in knowledge and support. Since we have measured knowledge and support before and after treatment, we can examine the causal links through a structural equation modeling (SEM) approach. While the classic mediation analyses methods can analyze the cases with only one mediator, the SEM method applied here allows us to examine the causal effects of two mediators. That is, we can examine the effect of advertisement type on curiosity and subsequently on increase in knowledge and support. The treatment variable in our analysis is “type of the advertisement” which has three categories (positive, negative, or no ads). The estimation method, however, can only be used with nominal variables with no more than two categories. We thus transform the treatment variable into two binary variables and estimate the model twice. First, we only consider the observations in the negative and control group to estimate the effect of negative advertisements as compared with the control group. Second, we only consider the observations in the positive and control groups to estimate the effects of positive advertisements as compared with the control group. The results are reported in Table 5. As reported in column 1, the curiosity of the sample of individuals in the US who were exposed to negative advertisements about ACA was 0.53 units higher than those who were not exposed to any kind of advertisements. The increased knowledge among these individuals is 1.55 units higher than those in the control group. Also, their increased level of support is 0.49 units higher than those that were not exposed to any kind of advertisements. As reported in column 2, those who watched positive advertisements were .32 units more curious than those who did not. However, the level of curiosity among them was less than that of individuals who watched negative advertisements (0.53 vs 0.32). The subsequent increase in knowledge of the individuals in the positive group about ACA was 0.76 units higher than those in the control group. Again, the control group shows lower levels of increased knowledge when compared with the negative group (1.55 vs 0.76). The increased level of support for ACA among the individuals who watched positive advertisement is not statistically different from those who did not watch any kind of advertisements. The same pattern of effects can be observed among the individuals outside of the US (columns 3 and 4). The results are similar for advertisements about Common Core education standards among individuals both inside US (columns 5 and 6) and outside the US (columns 7 and 8). Comparing the effects of positive and negative advertisements across all the samples and topics show that negative advertisements consistently have much stronger effects in arousing the curiosity of individuals.

**Table 5:** Path coefficients.

Dependent Variable / Independent Variable	ACA				Common Core Standards			
	Inside USA		Outside USA		Inside USA		Outside USA	
	(1)	(2)	(3)	(4)	(5)	(6)	(7)	(8)
***Support Increase***								
Knowledge Increase	0.49*** (0.12)	0.06 (0.13)	0.29*** (0.05)	0.27*** (0.05)	0.42*** (0.09)	0.15 (0.13)	0.31*** (0.05)	0.17*** (0.04)
***Knowledge Increase***								
Curiosity	1.55*** (0.35)	0.76*** (0.07)	2.45*** (0.38)	2.67*** (0.31)	1.49*** (0.36)	0.55*** (0.10)	3.01*** (0.33)	2.00*** (0.48)
***Curiosity***								
Exposure to negative ads vs. no ads	0.53*** (0.11)		0.56*** (0.13)		0.39** (0.13)		0.69*** (0.09)	
Exposure to positive ads vs. no ads		0.32** (0.11)		0.11 (0.13)		0.10 (0.14)		0.42*** (0.12)
***Fit Indices***								
*χ*^2^,(*df*)	144.26,(6)		77.15,(6)		20.62,(6)		260.61,(6)	
NFI	0.86		0.90		0.97		0.94	
NNFI	0.75		0.87		1.33		0.89	
CFI	0.87		0.93		1.00		0.95	

p<0.05

** p<01

*** p<0.001

Interestingly, curiosity of individuals outside of the US leads to much higher levels of increase in their knowledge about both topics. For example, as shown in column 1, the level of knowledge of individuals in the US who watched negative advertisements about ACA increases by 1.55 units, while as shown in column 3, the level of knowledge about ACA among their counterparts outside of the US increases by 2.45. Another interesting finding is that for individuals in the US, in the group who watched negative advertisements, increased knowledge has a more salient effect on increasing the level of support for both of the topics as compared to the ones who watched positive advertisements. For example, as shown in column 1, increased knowledge among those inside the US, leads to .49 units of increase in support for those who watched negative advertisements, while as shown in column 2, this size of this effect is only 0.06 and not statistically significant for those who watched positive advertisements. We observe similar effects for the topic of Common Core education standards as shown in columns 5 and 6. However, for individuals outside of the US, the magnitude of the effect of increased knowledge on increased support for both topics among those who were exposed to negative advertisements is not very much higher than on those who watched negative advertisements. For example, as shown in columns 3 and 4, respectively, increased support for ACA among samples outside of the US is 0.29 in negative group and 0.27 in positive group which are not statistically different. As shown in columns 7 and 8, the size of these effects are respectively equal to 0.31 and 0.17 in negative and positive groups.

The power of our tests would have been much higher with a larger sample size. Although our sample size is small, we have run four independent experiments (ACA in the USA, ACA outside of the USA, CC in the USA and CC outside of the USA) and the results of all four experiments are consistent with each other and confirm our hypotheses. This consistency of results diminishes the concerns over the power of tests of our hypotheses to some extent.

### 4.3. Limitations

The content of an advertisement and the emotions that it provoke can affect how individuals make decisions [35,36]. As Corrigan and Brader [37] note, “Ads are not merely negative or positive; they also appeal to a variety of emotions, evoke associations to various groups in society, and differ in the extent and nature of their issue content, to name just a few salient attributes. Potential effects also go beyond mobilizing or demobilizing turnout to include influencing what voters learn, and how they form opinions.” Since we have used real advertisements in our experiment, it would be extremely difficult for us to control for their content or the type of emotions they provoke. To overcome this issue, we have tried our best to choose video treatments that their content are as similar to each other as possible. Note that because these advertisements were created by different parties with different purposes and for different target groups, their content will inevitably be different from each other. For example, the Anti-ACA ads are more focused on creating a sense of fear from the involvement of government in the healthcare system while pro-ACA advertisements are more focused on highlighting the potential benefits of the law for citizens. Because of this limitation, our results in this experiment may be confounded by the contents of the advertisements.

The other limitation of this study is its measurement of curiosity. We have asked the respondents to choose from a list of available topics and because of the potential priming effects, they may have been inclined to choose the subject of ad, without much curiosity. In future studies, one could use more precise measures of curiosity; for example, one could ask the respondents to type a subject which they are interested to know more about instead of just choosing from the available option. In the current study, we could not implement such approach because we needed to automate the process for an online experiment in which the additional information are shown to the users based on their choices. This technical limitation prevented us from allowing users to type in any topic they preferred because we could only provide additional information on a limited set of topics. Finally, the other limitation of this study is its relatively small sample size which does not provide enough power to examine additional effects, such as differential partisan responses.

Finally, in a controlled setting, it is much easier to demonstrate and measure curiosity and information-seeking behavior that it is in the outside environment. Also, the cost of information seeking in our experiment is minimal as compared to the actual cost that potential ACA enrollees incur for independent acquisition of knowledge about insurances. The optimal research method to examine this question would have been a prospective cohort study in which a group of individuals would be followed to see how their behavior changes as a result of their exposure to negative advertisements. However, we had to limit ourselves to the experimental design and abandon the prospective design for two reasons. First, due to rarity of negative advertisements, it is very difficult to find an opportunity to conduct this research following actual observations outside of a research lab. Second, even if we have the opportunity to study the effects of negative advertisements outside of a controlled experiment, ensuring that exposure to negative advertisements is random and truly exogenous is very difficult. For example, in the case of ACA, some individuals would be more exposed to anti-ACA advertisements due to self-selecting certain media outlets based on their political views which are also correlated with their decision to purchase such insurances. A controlled experiment in which we can randomize the exposure to negative advertisements is therefore a tradeoff between the internal and external validity of our findings.

## 5. Discussion

Prior research has examined the effects of negative information on individuals’ opinions about subjects that do not require high cognitive processing. In this research we examine a situation in which the subject requires high cognitive processing and show that negative and rhetorical messages that are void of any substantial information will positively affect the individuals’ impressions about the subject. We also show that positive messages are not as effective as their negative counterparts and use the information gap theory to argue that negative messages are more likely to trigger the curiosity of individuals, and if the subject or idea is potentially beneficial for the individuals, they are more likely to support it based on the information they obtain independently.

Academic research has cast doubts on the old adage that “any publicity is good publicity” and has shown that negative publicity most often leads to negative outcomes. Our paper contributes to the literature by documenting another case of positive effects of exposure to negative information and examining other conditions under which these effects could be observed. Berger et al. [6] uncovered the moderating effect of awareness on the relationship between exposure to negative information and purchase decisions. The theoretical contribution of the current work is that it uncovers a mediated process through which the relationship between negative information and purchase decisions is sequentially mediated by curiosity and knowledge.

In this research, we analyzed the effects of anti-ACA advertisements and showed that these advertisements led to an increase in the ACA enrollment. To explain this relationship, we relied on the knowledge-gap theory and argued that exposure to negative information creates a knowledge-gap among individuals and leads them to fill it by seeking more information about the advertised subject. This will consequently lead to higher knowledge and support. For example, in the case of ACA, assuming that subsidized health insurances are rational economic choices for individuals, we expect the more knowledgeable individuals to also be more likely to enroll in ACA health insurances. To test our hypotheses, we designed an experiment. The measurement and comparison of curiosity, knowledge and support before and after being exposed to negative, positive, or no information allowed us to examine how exactly individuals are affected by different kinds of advertisements about different subject. The results of this study support our main contention that exposure to negative information triggers the curiosity of individuals much more than positive advertisements. Such increased curiosity will lead to higher shifts in knowledge and subsequent support of the subjects of the advertisements. By testing our hypothesis on both Common Core education standards and ACA, we showed that the effects are not limited to ACA context and hold across different subjects and populations.

## Supporting information

S1 Appendix (DOCX)
