# Supplementary material for: (How) did attack advertisements increase Affordable Care Act enrollments?
Source: PLoS One. 2020 Feb 19;15(2):e0228185. doi: 10.1371/journal.pone.0228185 (PMC7029848; doi:10.1371/journal.pone.0228185)
Supplement: S1 Appendix — (DOCX) [file pone.0228185.s001.docx]

**ONLINE APPENDIX**

**S1: The Contents of the political advertisements**

To investigate the contents of the advertisements that were run during the Congress midterm elections in 2014, we searched for the campaign videos that included the word “Obamacare” on Youtube.com. Our sample includes 50 advertisements that were run by either the House or the Senate candidates in the states of Arizona (2), Georgia (4), Idaho (1), Illinois (2), Kansas (3), Kentucky (1), Louisiana (2), Michigan (4), Missouri (1), Mississippi (2), Montana (2), North Carolina (4), Nebraska (1), New Hampshire (4), New Mexico (2), New York (2), Oregon (1), South Carolina (2), South Dakota (4), Tennessee (2), Texas (1), Virginia (1), and West Virginia (2). Although the sample is relatively small, it is reasonably representative of the advertisements that were run by candidates who participated in 2014 House and Senate midterm elections. Using the transcripts of these advertisements, we created a *links graph* which represents the collocate clusters of key words (Stéfan and Rockwell 2015) and a *word cloud* that represents the frequency of the key words used in advertisements. To create the word cloud, we removed the name of the candidates as well as the word “Obamacare” from the transcripts. These words are the most frequently used ones in the advertisements and including them in the text analysis will lead to a vague word cloud in which these frequent words are very large and the other words are very small and unclear.

As depicted in figure S1.a, the words *Obama, business, healthcare, repeal, vote* and *Washington* are the most frequently used words. With focusing on the negative implications of the ACA for small businesses, these advertisements promise to repeal it. The Links graph showed in figure S1.b sheds more light on the relationship between the words used in these advertisements. While the name of the republican endorser of the advertisements is associated with words such as jobs and values, the name of the democrat opponent is mostly described as a supporter of Obama. The link graph also confirms that the attacks on Obamacare have been focused on its potentially negative effects on small businesses.

In summary, our preliminary analysis reveals that the negative advertisements merely used the word Obamacare as a negative concept. Anchoring on the potentially negative consequences of Obamacare for small businesses, the republican candidates portrayed their democrat opponents as the supporters of president Obama and his plans for implementing Obamacare. The analysis on the contents of these advertisements suggests that the republican candidates assumed that the public already agrees that ACA is inherently a bad phenomenon and thus there is no need to present them with more arguments against it. By merely claiming that the Obamacare will hurt small businesses they based their political appeal to the public on promising their future constituencies to repeal Obamacare.

Table S1 shows the distribution of the contents of all political ads during 2014. Anti-ACA ads were aired five times more than the pro-ACA ads. Anti-ACA ads constituted the major part of the Republican campaigns, as 36% of all the ads aired by Republicans were attacking ACA. On the other hand, Democrats distanced themselves from the issue and only 7.5% of all ads that were aired by them were about ACA.

|  | **Republicans** | | | **Democrats** | | |
| --- | --- | --- | --- | --- | --- | --- |
|  | *Only Anti-ACA* | *Anti-ACA and Other issues* | *Other issues* | *Only Pro ACA* | *Pro ACA and other issues* | *Other issues* |
| **House** | 15716 | 86075 | 208803 | 840 | 28040 | 285560 |
| **Senate** | 52635 | 145313 | 318021 | 5043 | 26856 | 457375 |

*Table S1: Summary of the contents of ads aired during the midterm elections in 2014*

**S2: Rationale for Measuring the State Characteristics**

Although the states had the option to expand their Medicaid program, many of them decided not to do so. Most of the states that did not expand their Medicaid program also refused to set up their own insurance exchange platforms. These states generally lean towards the political right. It is more difficult to enroll in ACA in states with such characteristics. In our analysis, *Medicaid_expansion* is a dummy variable which is equal to one if a state has expanded its Medicaid program under ACA and is equal to zero otherwise. Similarly, *State_exchange* is a dummy variable which is equal to one if the state has established its own insurance exchange platform. *Liberal_voters* variable measures the percentage of the democrat votes in 2012 presidential election. Religious beliefs may also affect the ACA enrollment ratio. In particular, the Catholic Church opposes certain provisions of the ACA which pertain to coverage of sterilizations and abortion drugs and procedures. We expect the states in which the Catholic Church has more influence to experience lower enrollment ratios. *Catholic_Church* variable, as a proxy for the influence of the church, represents the percentage of Catholic Church adherents among the adult population of a state.

*Education* represents the percentage of college educated population in each state. The higher percentage of college educated population indicate better overall economic prosperity of a state which results in higher employer sponsored insurance coverage and lower demand for ACA. Higher education also represents higher concerns over privacy. Educated people may be more concerned with releasing their financial and personally identifiable data to the insurance exchanges, and these concerns may have been elevated even more by the serious technical glitches of the websites.

Previous studies (Flynn et al. 1994) show that males perceive health risks as much smaller and much more acceptable than females. This implies that women would be more willing to mitigate the financial burden of the health risks by enrolling in the ACA. The variable *Uninsured_females* represents the percentage of females among the uninsured population of a state

Prior studies and speculations indicated that Latinos are much less likely to enroll in ACA insurance plans due to lack of awareness, language barriers and concerns by mixed-status families (Luhby) *Uninsured_latinos* represents the percentage of Latinos among the uninsured population of a state. We expect the *Uninsured_females* and *Uninsured_latinos* to have opposite effects on ACA enrollment. While the former drives the enrollment ratio up, the latter drives it down.

Since the utility of having health insurance is lower for individuals with better overall health condition, we expect the young adults to be less likely to purchase insurance. The *Young_invincibles* variable represents the percentage of uninsured adults who are younger than 34 years old in each state. We expect the states with higher percentage of *Young_invincibles* to experience lower enrolment ratios. *Low_income* variable represents for the percentage of uninsured adults with income lower than 134% of federal poverty line. Such adults are the main target population for the ACA insurances and thus we expect the *Low_income* to be positively associated with higher ACA enrolment ratios. We also consider the cost of enrollment in ACA plans as well as the cost of the private insurance alternatives before the ACA implementation. *Log (Private Insurance Premium)* and *Log (ACA premium)* respectively represent the logarithm of the average cost of the premiums for private insurances before the ACA implementation and the average of lowest cost silver, second lowest cost silver and lowest cost bronze plans of ACA insurance options after its implementation in each state. We expect the enrollment ratios to be positively associated with the *Log (Private Insurance Premium)* and negatively associated with *Log (ACA premium)*. Finally, *Insurance_cancelations* represents the proportion of individuals who had previously purchased private insurances which were cancelled after ACA implementation. Since such individuals are more willing and probably have more means to purchase an alternative insurance policy at the ACA marketplaces, we expect *Insurance_cancelations* to positively affect enrollment ratios.

**S3. Derivation of Equation (1)**

Since the enrollment ratio $y_{i}$ falls within the range of$\left( 0,1 \right)$, we can assume that it has a beta distribution with shape parameters $\omega$ and $\tau$ such that

$$f\left( y; \omega,\tau\right)=\frac{\Gamma\left( \omega+\tau\right)}{\Gamma\left( \omega\right)\Gamma\left( \tau\right)}y^{\omega-1}\left( 1-y \right)^{\tau-1} (a.0)$$

where $\Gamma\left( . \right)$ denotes the gamma function.

The mean and variance of the beta distribution are respectively

$\mu=E\left( y \right)=\frac{\omega}{\omega+\tau} (a.1)$

$$\sigma^{2}=Var\left( y \right)=\frac{\omega\tau}{\left( \omega+\tau\right)^{2}\left( \omega+\tau+1 \right)}=\frac{\mu(1-\mu)}{\left( \omega+\tau+1 \right)} (a.2)$$

if we define $\phi=\omega+\tau$ then we can re-write eq $(a.1)$ as

$$\omega=\mu\left( \omega+\tau\right)\underset{\to}{\phi=\omega+\tau}\omega=\mu\phi\left( a.3 \right)$$

$$\tau=\frac{\omega(1-\mu)}{\mu}\underset{\to}{\omega=\mu\phi}\tau=\phi-\mu\phi\left( a.4 \right)$$

We can also re-write eq $\left( a.2 \right)$ as

$$\sigma^{2}=\frac{\mu(1-\mu)}{\left( \phi+1 \right)} \left( a.5 \right)$$

Equation $(a.5)$ implies that as $\phi$ decreases, the variance increases.

The above transformation allows us to consider $\mu$ as a location parameter and $\phi$ as a precision parameters. By estimating $\omega$ and $\tau$, we are effectively estimating $\mu$ and $\phi$.

If we let $\boldsymbol{X}$ and $\boldsymbol{W}$ be the matrices of the variables that affect $\mu$ and $\phi$, then we can use the logit and log links respectively to define $\mu$ and $\phi$ as functions of $\boldsymbol{x}_{\boldsymbol{i}}$and $\boldsymbol{w}_{\boldsymbol{i}}$**.**

$$\mathrm{Ln}\left( \frac{\mu_{i}}{1-\mu_{i}} \right)=\boldsymbol{x}_{\boldsymbol{i}}\beta\left( a.6 \right)$$

$$\mathrm{Ln}\left( \phi_{i} \right)=-\boldsymbol{w}_{\boldsymbol{i}}\delta\left( a.7 \right)$$

Note that since $\mu$ is the mean of the beta distribution and by definition has to be in the interval of 0 and 1, we use the logit link function to map the real line onto the open unit interval. Similarly, the log link function is chosen to make sure that $\phi$ is strictly positive.

Inversing the link functions above gives the location and dispersion sub-models as

$$\mu_{i}=\frac{exp(\boldsymbol{x}_{\boldsymbol{i}}\beta)}{1+exp(\boldsymbol{x}_{\boldsymbol{i}}\beta)} \left( a.8 \right)$$

$$\phi_{i}=exp(-\boldsymbol{w}_{\boldsymbol{i}}\delta) \left( a.9 \right)$$

Based on eq. $(a.0)$, we define the log-likelihood function for observation $y_{i}$ as

$$\mathrm{Ln} L\left( \omega,\tau,y_{i} \right)=\mathrm{Ln}\Gamma\left( \omega+\tau\right)-\mathrm{Ln}\Gamma\left( \omega\right)-\mathrm{Ln}\Gamma\left( \tau\right)+\left( \omega-1 \right){Ln(y}_{i})+(\tau-1)\mathrm{Ln}\left( 1-y_{i} \right) \left( a.10 \right)$$

Substituting equations $(a.3)$ and $(a.4)$ in equation $(a.10)$ we will have

$\mathrm{Ln} L\left( \mu, \phi,y_{i} \right)=\mathrm{Ln}\Gamma\left( \phi\right)-\mathrm{Ln}\Gamma\left( \mu\phi\right)-\mathrm{Ln}\Gamma\left( \phi-\mu\phi\right)+\left( \mu\phi-1 \right){Ln(y}_{i})+(\phi-\mu\phi-1)\mathrm{Ln}\left( 1-y_{i} \right)$ $\left( a.11 \right)$

And finally substituting equations $(a.8)$ and $(a.9)$ in equation $(a.11)$ we will have

$$\mathrm{Ln} L\left( \beta,\delta,y_{i},\boldsymbol{X}, \boldsymbol{W} \right)=\mathrm{Ln}\Gamma\left( \exp\left( -\boldsymbol{w}_{\boldsymbol{i}}\delta\right) \right)-\mathrm{Ln}\Gamma\left( \frac{\exp\left( \boldsymbol{x}_{\boldsymbol{i}}\beta-\boldsymbol{w}_{\boldsymbol{i}}\delta\right)}{1+\exp\left( \boldsymbol{x}_{\boldsymbol{i}}\beta\right)} \right)-\mathrm{Ln}\Gamma\left( \frac{\exp\left( -\boldsymbol{w}_{\boldsymbol{i}}\delta\right)}{1+\exp\left( \boldsymbol{x}_{\boldsymbol{i}}\beta\right)} \right)+\left( \frac{\exp\left( \boldsymbol{x}_{\boldsymbol{i}}\beta-\boldsymbol{w}_{\boldsymbol{i}}\delta\right)}{1+\exp\left( \boldsymbol{x}_{\boldsymbol{i}}\beta\right)}-1 \right){Ln(y}_{i})+(\frac{\exp\left( -\boldsymbol{w}_{\boldsymbol{i}}\delta\right)}{1+\exp\left( \boldsymbol{x}_{\boldsymbol{i}}\beta\right)}-1)\mathrm{Ln}\left( 1-y_{i} \right) \left( a.12 \right)$$

The maximum likelihood method allows us to estimate the coefficients of $\beta and \delta$ given the sample of$y_{i},\boldsymbol{X}, \boldsymbol{W}$**.**

Maximum Likelihood Estimation (MLE) with a beta distributed dependent variable, suggested by (33) is a suitable method for instances in which the dependent variable is bounded and the homoscedasticity assumption is not necessarily held true. In our context, the dependent variable is the enrollment ratio which is bounded between 0 and 1. Moreover, we have cannot be certain that the error terms are homoscedastic. These conditions warrant the implementation of an MLE method with a beta dependent variable. The other advantage of our econometric specification and estimation method is that it allows us to study the effects of model covariates on not only the location of the dependent variable but also on its variability. This implies that, we can examine how the covariates predict the mean and dispersion of enrollment ratios. An alternative approach to beta regression would be a logit function which maps the ratio values to the real line. Although this method would solve the problem of bounded dependent variable, it still has multiple drawbacks. First, the coefficient estimates would be difficult to interpret in terms of the original response. Second, this method assumes homoscedastic error terms and is not suitable for the data involving unit interval which are typically heteroscedastic with higher variation more around the mean, and less around the two ends of the unit interval. Third, the proportion measures are asymmetric and thus inferences based on the normality assumption can be misleading.

Let 𝑦 denote the ACA enrollment ratio and $i=1,\ldots,51$ index the fifty states plus the District of Columbia. Assume that $y_{i}$ has a *beta* distribution with shape parameters $\omega$ and$\tau$. The first and second moments of the beta distribution are $\mu=\frac{\omega}{\omega+\tau}$ and$\sigma^{2}=\frac{\mu(1-\mu)}{\left( \omega+\tau+1 \right)}$. If we define$\phi=\omega+\tau$, then we can show that $\omega=\mu\phi$ and$\tau=\phi-\mu\phi$. This re-parametrization helps us to redefine the beta distribution and its corresponding log likelihood function in terms of the location $(\mu)$ and dispersion $(\phi)$ parameters. Using the logit and log link functions to respectively regress $\mu$ and $\phi$ on matrices of the variables $\boldsymbol{X}$ and$\boldsymbol{W}$, we have $\mu_{i}={exp(\boldsymbol{x}_{\boldsymbol{i}}\beta)}/\left[ 1+exp(\boldsymbol{x}_{\boldsymbol{i}}\beta) \right]$ and $\phi_{i}=exp(-\boldsymbol{w}_{\boldsymbol{i}}\delta)$ and thus can write the log likelihood function, $\mathrm{Ln} L\left( \beta,\delta,y_{i},\boldsymbol{X}, \boldsymbol{W} \right)$ as follows:

$$\left( 1 \right)\mathrm{Ln} L\left( \beta,\delta,y_{i},\boldsymbol{X}, \boldsymbol{W} \right)=\mathrm{Ln}\Gamma\left( \exp\left( -\boldsymbol{w}_{\boldsymbol{i}}\delta\right) \right)-\mathrm{Ln}\Gamma\left( \frac{\exp\left( \boldsymbol{x}_{\boldsymbol{i}}\beta-\boldsymbol{w}_{\boldsymbol{i}}\delta\right)}{1+\exp\left( \boldsymbol{x}_{\boldsymbol{i}}\beta\right)} \right)-\mathrm{Ln}\Gamma\left( \frac{\exp\left( -\boldsymbol{w}_{\boldsymbol{i}}\delta\right)}{1+\exp\left( \boldsymbol{x}_{\boldsymbol{i}}\beta\right)} \right)+\left( \frac{\exp\left( \boldsymbol{x}_{\boldsymbol{i}}\beta-\boldsymbol{w}_{\boldsymbol{i}}\delta\right)}{1+\exp\left( \boldsymbol{x}_{\boldsymbol{i}}\beta\right)}-1 \right){Ln(y}_{i})+(\frac{\exp\left( -\boldsymbol{w}_{\boldsymbol{i}}\delta\right)}{1+\exp\left( \boldsymbol{x}_{\boldsymbol{i}}\beta\right)}-1)\mathrm{Ln}\left( 1-y_{i} \right)$$

Maximizing the above log likelihood function gives the estimates of $\beta$ and $\delta$, and allows us to study how the set of covariates $\boldsymbol{X}$ and$\boldsymbol{W}$ affect the mean and variability of the enrollment ratios in different states.

Both $\boldsymbol{X}$ and$\boldsymbol{W}$ matrices comprise of a set of four principle components derived from a principle component analysis of the state level characteristics (*PC1* to *PC4*), pro-ACA advertisements (*Pro ACA Ads*), along with our main endogenous independent variable (*Anti ACA Ads*) in each state.

**S4: Principle Component Analysis**

As shown in Table 2 in the main paper, many of the variables representing states’ characteristics are highly correlated with each other. More importantly, these variables may be driven by common yet unobserved factors which are impossible to quantify. For example, education policies of a state and its tax incentives for college education may result in both higher ratios of college educated adults, and higher involvement of females in labor market. Both of these effects will result in higher employment ratios and thus employment sponsored insurance. While we are not able to effectively quantify the states’ education policies, we can proxy it with such observed characteristics.

| **Component** | **Initial eigenvalues** | | | **Rotation sums of squared loadings** | | |
| --- | --- | --- | --- | --- | --- | --- |
|  | **Eigenvalue** | **% of variance** | **Cumulative %** | **Total** | **% of variance** | **Cumulative %** |
| 1 | 3.8827 | 0.3236 | 0.3236 | 3.4817 | 0.2901 | 0.2901 |
| 2 | 1.7475 | 0.1456 | 0.4692 | 1.6904 | 0.1409 | 0.431 |
| 3 | 1.3123 | 0.1094 | 0.5785 | 1.5154 | 0.1263 | 0.5573 |
| 4 | 1.1112 | 0.0926 | 0.6711 | 1.3662 | 0.1138 | 0.6711 |
| 5 | 0.992 | 0.0827 | 0.7538 |  |  |  |
| 6 | 0.735 | 0.0613 | 0.8151 |  |  |  |
| 7 | 0.5985 | 0.0499 | 0.8649 |  |  |  |
| 8 | 0.5468 | 0.0456 | 0.9105 |  |  |  |
| 9 | 0.4289 | 0.0357 | 0.9462 |  |  |  |
| 10 | 0.2863 | 0.0239 | 0.9701 |  |  |  |
| 11 | 0.2379 | 0.0198 | 0.9899 |  |  |  |
| 12 | 0.1209 | 0.0101 | 1 |  |  |  |

*Table S2: Total variance explained by components (Using Varimax rotation)*

We use PCA to reduce the number of the variables into a set of principal components. Each component is a linear combination of optimally weighted observed variables. More specifically, for observation $i$ we define the $j$th component, $C_{ij}$, such that $C_{ij}$=$\sum_{k=1}^{12} b_{kj}X_{ik}$ in which $X_{ik}$ represents the value of the $k$th variable for $i$th observation and $b_{kj}$ represents the optimal weight of $X_{ik}$. The weights $b_{kj}$ are optimal in the sense that for a given dataset, no other set of weights could produce a set of components that are more successful and accurate in accounting for variance in observed variables.

It is difficult to theorize the loadings of various variables on the principle components as reported in Table S3. In the following we discuss the reasons why the loadings do not necessarily need to have a theoretical explanation.

First, rather than creating a measurement model for a latent variable, our goal in this part of the analysis was to reduce the data by creating one or more index variables from a larger set of measured variables, we therefore undertook a PC analysis instead of the Factor Analysis. We used PC analysis to merely find optimal ways of combining variables into a small number of subsets, rather than identifying the structure underlying such variables or to estimate scores to measure latent factors themselves. Since in the PC analysis the components are simply geometrical abstractions, they do not map easily onto real world phenomena and therefore it is very difficult to interpret the loadings.

The second reason for the seemingly contradictory loadings in the PCA is the fact that we use state level data to estimate our PC regressions. The estimates driven from aggregated group data are not necessarily equal to those driven from individual level data. At the individual level, we can construct a principle component *(*$c$*)* such that $c_{i}$*=*$\beta x_{i}+u_{i}$ in which $x_{i}$ is uncorrelated with the error term and therefore$cov\left[ u_{i},x_{i} \right]=0$. The same regression model at the aggregate level will then become $\sum_{i=1}^{N} c_{i}$=$\beta\sum_{i=1}^{N} x_{i}+\sum_{i=1}^{N} u_{i}$. The estimates from the two models will be the same only if$cov\left[ \sum_{i=1}^{N} u_{i},\sum_{i=1}^{N} x_{i} \right]=0$. Although at the individual level, the error terms are uncorrelated with the repressors, there is no reason to assume that the same relationship will continue to hold at the aggregate level. This is due to the fact that the covariance of two aggregated variables depends on the covariance of the two variables within the same set of individuals as well as the covariance of the two variables between different individuals. This is formally defined as$cov\left[ \sum_{i=1}^{N} u_{i},\sum_{i=1}^{N} x_{i} \right]=\sum_{i=1}^{N} cov\left[ u_{i},x_{i} \right]+\sum_{i=1}^{N} \sum_{j\neq i} cov\left[ u_{j},x_{i} \right]$. There is no theoretical reason to assume that$\sum_{i=1}^{N} \sum_{j\neq i} cov\left[ u_{j},x_{i} \right]=0$, and therefore we cannot assume that$cov\left[ \sum_{i=1}^{N} u_{i},\sum_{i=1}^{N} x_{i} \right]=0$. While it is possible to expect a certain relationship between the loadings of the variables on the components driven from a PC analysis on individual level data, there is no reason to expect to observe the same relationship between the loadings of the components on the PC analysis of the aggregated data.

The difference between the individual and group level effect of income on tendency to vote Republican (Democrat) is a good practical example of our theoretical discussions. At the state level, the five poorest states per average income level (Mississippi, Arkansas, West Virginia, Alabama and Kentucky) all voted Republican while the five richest states per average income (Maryland, District of Columbia, New Jersey, Massachusetts and Connecticut) all voted Democrat. While at the state level, income negatively (positively) affects the tendency to vote Republican (Democrat), at the individual level the relationship is exactly the opposite such that the individuals with higher (lower) income are more likely to vote Republican (Democrat).

| Variable | Component | | | |
| --- | --- | --- | --- | --- |
|  | 1 | 2 | 3 | 4 |
| Medicaid Expansion | **0.68** | 0.20 | 0.18 | 0.31 |
| Education | **0.82** | 0.08 | 0.12 | -0.23 |
| State-run Exchange | **0.66** | 0.28 | 0.41 | 0.15 |
| Liberal voters | **0.85** | 0.09 | 0.05 | 0.03 |
| Uninsured Females | **-0.74** | -0.02 | 0.30 | 0.04 |
| Catholic church members | **0.60** | 0.44 | -0.28 | 0.21 |
| Uninsured Latinos | 0.08 | **0.87** | 0.28 | -0.06 |
| Log (Private Insurance Premium) | 0.27 | **0.74** | -0.28 | 0.01 |
| Insurance cancelations | 0.20 | -0.20 | **0.70** | -0.04 |
| Log (ACA premium) | 0.07 | -0.12 | **-0.58** | -0.14 |
| Low Income | -0.36 | 0.09 | 0.39 | **0.61** |
| Young Invincibles | 0.21 | -0.08 | -0.01 | **0.87** |

*Table S3: Rotated factor matrix (loading) of Principle Components*

**S5: Details of the exogeneity and relevance assumptions for the Instrumental Variable**

To show that competitiveness of the midterm elections empirically satisfies both exogeneity and relevance assumptions, we first run a regression model with a beta specification and study the effects of competitive index, *CI*, on the ACA enrollment ratio. As presented in Table S4, the instrumental variable has no effect on the location of the ACA enrollment ratio ($\mu$). This provides some preliminary empirical support that *CI* satisfies the exogeneity condition. There is no formal statistical method for testing the exogeneity condition in cases where there is only one candidate for instrumental variable, and we should only rely on theoretical justifications for the choice of the instrumental variable.

| **Variable** |  | |  | | **ACA enrollment ratio** | | |
| --- | --- | --- | --- | --- | --- | --- | --- |
|  | **(1)** | **(2)** | | **(3)** | | **(4)** | **(5)** |
| CI | 0.001520  (0.009802) | 0.001926  (0.009863) | | 0.009342  (0.008657) | | 0.008777  (0.008647) | 0.01051  (0.008600) |
| Anti-ACA Ads |  | -0.0000045  (0.000013) | |  | | 0.000004  (0.000008) | 0.000017  (0.000012) |
| Pro-ACA Ads |  | -0.0000037  (0.000056) | | -0.00002  (0.008657) | |  | -0.00007  (0.000049) |
| PC1 |  |  | | 0.08315  (0.05154) | | 0.09987  (0.05519) | 0.1109**  (0.05460) |
| PC2 |  |  | | -0.08734  (0.05375) | | -0.08483  (0.05408) | -0.07054  (0.05439) |
| PC3 |  |  | | -0.1277**  (0.05076) | | -0.1336**  (0.05197) | -0.1473**  (0.05181) |
| PC4 |  |  | | -0.1603***  (0.05000) | | -0.1621**  (0.04987) | -0.1820***  (0.05124) |
| Constant | -1.2534***  (0.08599) | -1.2251***  (0.09920) | | -1.3000***  (0.08251) | | -1.3449***  (0.09117) | -1.3527***  (0.09005) |

**p<0.1; **p<0.05; ***p<0.001*

*Table S4: ACA enrollment ratios as a function of CI and other controls*

We re-estimate our models using an alternative measure of competitiveness index, ${CI}^{'},$ which is a binary variable that is equal to one if the competitiveness index of midterm elections in a state is higher than its average and zero otherwise. The estimates based on this alternative instrumental variable are very similar to the ones reported in the paper and further confirm the robustness of our current results. This additional instrumental variable also allows us to run the Sergan’s over-identifying restriction test (34) to further examine the appropriateness of our main instrumental variable (*CI*). The Sargan’s test statistic is $N\times R^{2}$when $N$ is number of observations and $R^{2}$is the coefficient of determination from regressing the residuals of the second stage regression onto the set of the exogenous variables. The statistic $N\times R^{2}$will have an asymptotic Chi-square distribution with $k$ degrees of freedom, where $k$ is the number of endogenous variables minus the number of instrumental variables which in our case will be 1. Under the null hypothesis, $N\times R^{2}$should be very small and rejection of the null hypothesis indicates that at least one of the instruments is not valid. In our context, The $R^{2}$ from regressing the residuals of the second stage of the 2SLS on the exogenous variables is equal to 0.0086. Since we have 51 observations, Sargen’s test statistic will be 0.4386. This leads to a p-value of 0.5078 and thus the null hypothesis of the Sargan’s test is not rejected which means that both instruments satisfy the exogeneity condition. Note that although both instruments are valid, we only use *CI* in our analysis as they are conceptually very similar.

**S6: Descriptive Statistics of the Experiment Respondents**

| **Variable** | **Inside USA** | | | **Outside USA** | | |
| --- | --- | --- | --- | --- | --- | --- |
|  | Control | Positive | Negative | Control | Positive | Negative |
| **Ethnicity** |  |  |  |  |  |  |
| American Indian or Alaskan Native | 1 | 0 | 0 | 1 | 0 | 1 |
| Asian or Pacific Islander | 2 | 2 | 2 | 15 | 12 | 13 |
| Black or African American | 0 | 1 | 2 | 2 | 1 | 0 |
| Hispanic or Latino | 1 | 1 | 0 | 0 | 2 | 3 |
| North African - Arab | 0 | 0 | 0 | 0 | 0 | 1 |
| White / Caucasian | 21 | 20 | 20 | 7 | 9 | 7 |
| Prefer not to answer | 0 | 1 | 1 | 0 | 1 | 0 |
| **Household Income** |  |  |  |  |  |  |
| $0-$24,999 | 3 | 7 | 6 | 12 | 13 | 12 |
| $25,000-$49,999 | 9 | 7 | 9 | 8 | 8 | 4 |
| $50,000-$74,999 | 10 | 5 | 6 | 3 | 1 | 6 |
| $75,000-$99,999 | 0 | 5 | 3 | 2 | 2 | 2 |
| $100,000-$124,999 | 3 | 1 |  | 0 | 0 | 1 |
| $125,000-$149,999 | 0 | 0 | 1 | 0 | 1 | 0 |
| $150,000 and above | 0 | 0 | 0 | 0 | 0 | 0 |
| **Gender** |  |  |  |  |  |  |
| Female | 14 | 11 | 10 | 11 | 6 | 6 |
| Male | 11 | 14 | 15 | 14 | 19 | 19 |
| **Education** |  |  |  |  |  |  |
| 4^th^ grade | 0 | 0 | 0 | 1 | 0 | 0 |
| 5^th^ grade | 0 | 0 | 0 | 1 | 0 | 0 |
| 8^th^ grade | 0 | 0 | 0 | 0 | 0 | 1 |
| 9^th^ grade | 0 | 0 | 0 | 0 | 0 | 0 |
| 11^th^ grade | 1 | 0 | 0 | 0 | 0 | 0 |
| Graduated from high school | 3 | 3 | 2 | 0 | 0 | 2 |
| 1 year of college | 0 | 4 | 1 | 0 | 1 | 1 |
| 2 years of college | 3 | 3 | 5 | 1 | 3 | 1 |
| 3 years of college | 2 | 2 | 3 | 0 | 1 | 6 |
| Graduated from college | 13 | 13 | 7 | 14 | 12 | 10 |
| Some graduate school | 0 | 0 | 4 | 1 | 1 | 1 |
| Completed graduate school | 3 | 0 | 3 | 7 | 7 | 3 |
| **Marital Status** |  |  |  |  |  |  |
| Divorced | 1 | 1 | 3 | 0 | 0 | 0 |
| In a domestic partnership or civil union | 0 | 2 | 0 | 2 | 1 | 1 |
| Married | 13 | 7 | 4 | 11 | 9 | 9 |
| Single, but cohabiting with another | 1 | 0 | 1 | 4 | 1 | 3 |
| Single, never married | 1 | 1 | 4 | 8 | 14 | 11 |
| Widowed | 9 | 14 | 13 | 0 | 0 | 1 |
| **Political Ideology** |  |  |  |  |  |  |
| Extremely conservative | 3 | 0 | 0 | 1 | 0 | 0 |
| Slightly conservative | 6 | 6 | 2 | 2 | 5 | 5 |
| Neither liberal nor conservative | 5 | 4 | 6 | 6 | 8 | 8 |
| Slightly liberal | 9 | 10 | 12 | 12 | 10 | 10 |
| Extremely liberal | 2 | 5 | 5 | 4 | 2 | 2 |
| **Age (Average)** | 37.24 | 35.72 | 35.6 | 29.72 | 30.44 | 29.28 |

*Table S5: Sample characteristics of participants in the ACA advertisements experiment*

| **Variable** | **Inside USA** | | | **Outside USA** | | |
| --- | --- | --- | --- | --- | --- | --- |
|  | Control | Positive | Negative | Control | Positive | Negative |
| **Ethnicity** |  |  |  |  |  |  |
| American Indian or Alaskan Native | 0 | 0 | 1 | 1 | 1 | 1 |
| Asian or Pacific Islander | 2 | 2 | 5 | 7 | 13 | 10 |
| Black or African American | 2 | 1 | 3 | 1 | 0 | 1 |
| Hispanic or Latino | 2 | 1 | 2 | 5 | 2 | 6 |
| North African - Arab | 0 | 0 | 0 | 0 | 0 | 0 |
| White / Caucasian | 19 | 21 | 14 | 11 | 9 | 7 |
| Prefer not to answer | 0 | 0 | 0 | 0 | 0 | 0 |
| **Household Income** |  |  |  |  |  |  |
| $0-$24,999 | 5 | 5 | 7 | 9 | 10 | 11 |
| $25,000-$49,999 | 6 | 8 | 6 | 9 | 8 | 5 |
| $50,000-$74,999 | 10 | 8 | 6 | 4 | 2 | 4 |
| $75,000-$99,999 | 1 | 2 | 2 | 0 | 5 | 5 |
| $100,000-$124,999 | 2 | 1 |  | 0 | 0 | 0 |
| $125,000-$149,999 | 0 | 0 | 2 | 1 | 0 | 0 |
| $150,000 and above | 1 | 1 | 2 | 2 | 0 | 0 |
| **Gender** |  |  |  |  |  |  |
| Female | 10 | 9 | 10 | 12 | 9 | 6 |
| Male | 15 | 16 | 15 | 13 | 16 | 19 |
| **Education** |  |  |  |  |  |  |
| 4^th^ grade | 0 | 0 | 0 | 1 | 0 | 0 |
| 5^th^ grade | 0 | 0 | 0 | 0 | 0 | 0 |
| 8^th^ grade | 0 | 0 | 0 | 0 | 0 | 0 |
| 9^th^ grade | 0 | 0 | 0 | 1 | 0 | 0 |
| 11^th^ grade | 0 | 1 | 0 | 0 | 0 | 0 |
| Graduated from high school | 1 | 0 | 2 | 1 | 1 | 0 |
| 1 year of college | 3 | 2 | 1 | 2 | 2 | 0 |
| 2 years of college | 7 | 3 | 6 | 2 | 1 | 3 |
| 3 years of college | 0 | 3 | 2 | 1 | 4 | 2 |
| Graduated from college | 9 | 11 | 10 | 12 | 10 | 15 |
| Some graduate school | 1 | 1 | 2 | 1 | 1 | 0 |
| Completed graduate school | 4 | 4 | 2 | 4 | 6 | 5 |
| **Marital Status** |  |  |  |  |  |  |
| Divorced | 2 | 1 | 2 | 0 | 1 | 0 |
| In a domestic partnership or civil union | 0 | 1 | 1 | 2 | 1 | 2 |
| Married | 13 | 7 | 5 | 11 | 9 | 10 |
| Single, but cohabiting with another | 0 | 0 | 0 | 1 | 2 | 0 |
| Single, never married | 2 | 0 | 5 | 11 | 12 | 13 |
| Widowed | 7 | 16 | 11 | 0 | 0 | 0 |
| **Political Ideology** |  |  |  |  |  |  |
| Extremely conservative | 0 | 3 | 1 | 0 | 1 | 0 |
| Slightly conservative | 5 | 5 | 4 | 4 | 2 | 4 |
| Neither liberal nor conservative | 5 | 6 | 3 | 4 | 8 | 5 |
| Slightly liberal | 11 | 7 | 10 | 12 | 10 | 14 |
| Extremely liberal | 4 | 4 | 7 | 5 | 4 | 2 |
| **Age (Average)** | 39.16 | 37.56 | 32.48 | 33.04 | 31.24 | 31.6 |

*Table S6: Sample characteristics of participants in the Common Core Standards advertisements experiment*

The overall design of the trial with CC topic is the same as the ACA trial, the difference is only in the topic as explained in the following. After random assignment in the first step, in the second step we provide a quiz about CC to measure the pre-exposure knowledge of respondents about CC. We also ask individuals to indicate their level of support about CC (rather than ACA). In the third step, positive and negative groups are exposed to respectively, positive and negative ads about CC, rather than ACA. To measure the curiosity in the fourth step, we consider an individual curious if he opts to know more about CC and incurious otherwise. Similar to the ACA experiment, in the fifth step, individuals read a paragraph about the topic that they chose in the previous step. Finally, in the sixth step, we repeat the questions of the second step to measure the knowledge and support of individuals about CC after they have been exposed to the treatments.


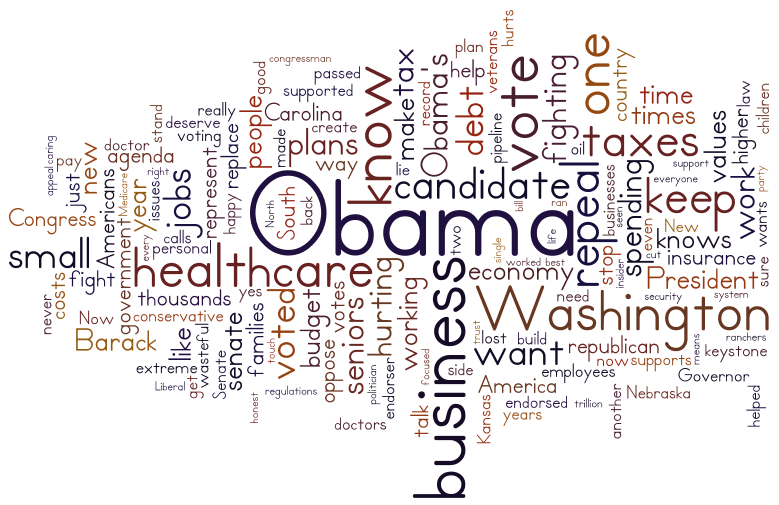


*Figure S1.a: The word cloud of the transcripts of the anti-ACA advertisements*


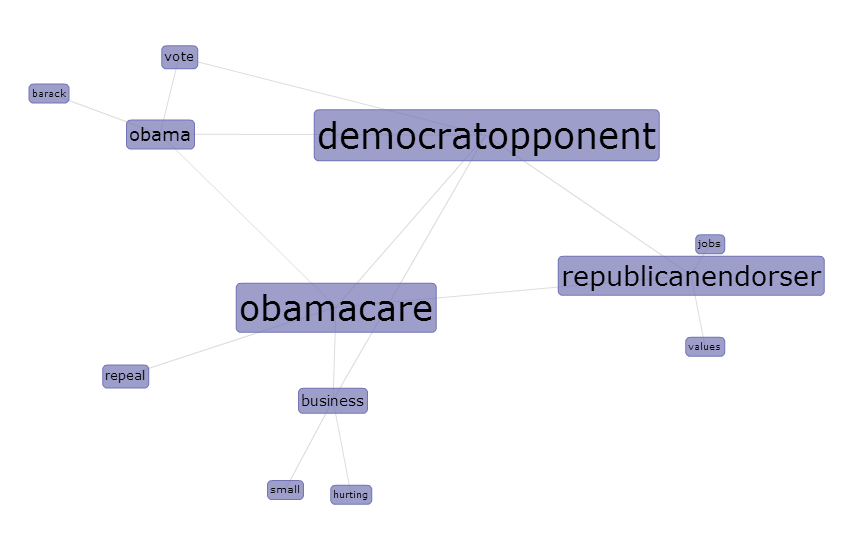


*Figure S1.b: The link graph of the transcripts of the anti-ACA advertisements*

*
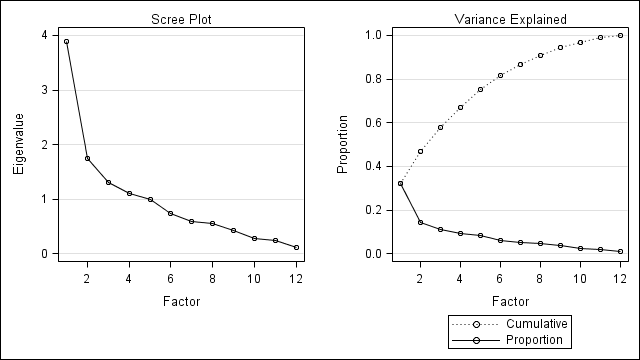
*

*Figure S2: Scree and variance plot*


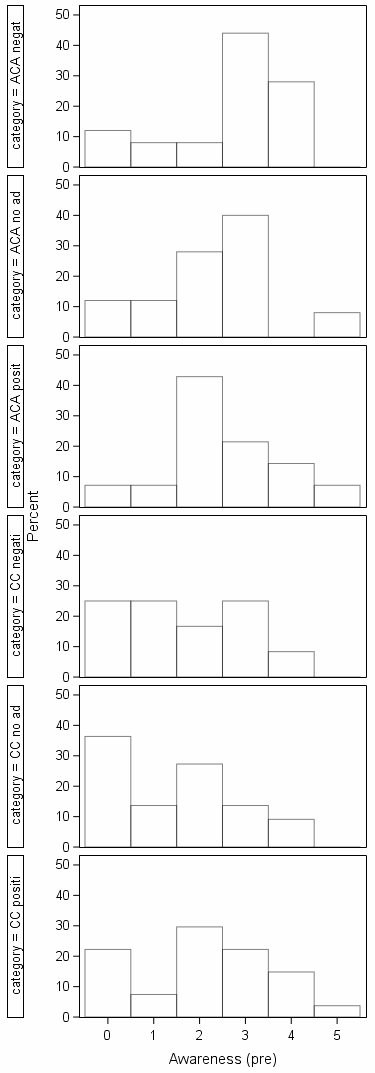

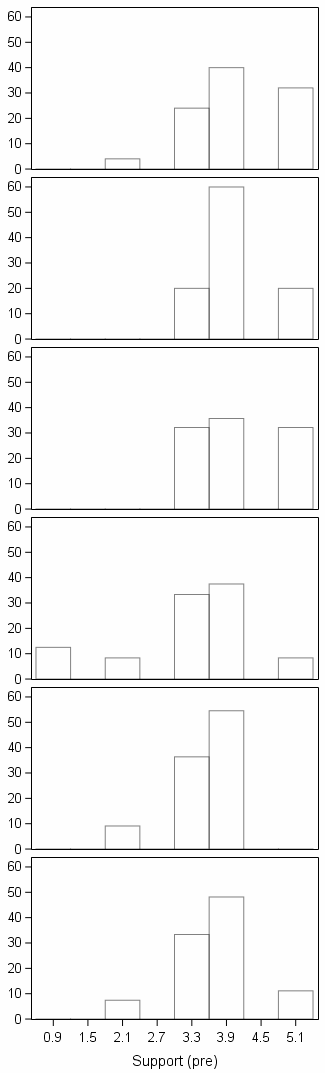


*Figure S3: Distribution of baseline of Knowlage (awareness) and Support of ACA and CC among respondants inside the US*


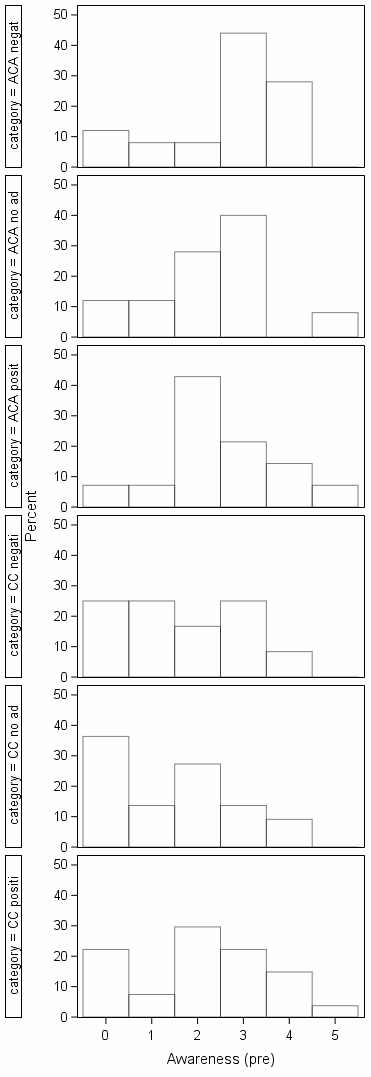

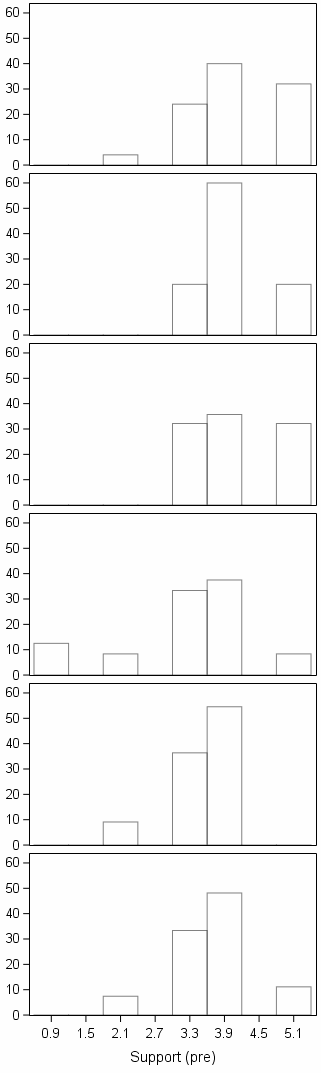


*Figure S4: Distribution of baseline of Knowlage (awareness) and Support of ACA and CC among respondants outside the US*
